# Localization of T and B Lymphocytes to the White Pulp of the Spleen is Independent of L-, E-, and P-Selectin

**DOI:** 10.1100/tsw.2003.42

**Published:** 2003-06-09

**Authors:** Mitchell H. Grayson, David D. Chaplin

**Affiliations:** Division of Allergy and Immunology, Department of Internal Medicine, Washington University School of Medicine, Campus Box 8122, 660 S. Euclid Avenue, St. Louis, MO 63110, USA

**Keywords:** spleen, lymphocytes, migration, selectin

## Abstract

T and B cell interactions are thought to be of prime importance in the generation of a humoral immune response. These interactions are thought to take place in the secondary lymphoid organs. The largest of which is the spleen. While the pathways involved in lymphocyte migration into other secondary lymphoid organs have been unraveled, very little is understood about T and B cell migration to the spleen. We report that adoptively transferred T lymphocytes appear more rapidly within the lymphoid compartment of the spleen than do B lymphocytes. Indeed, half of the transferred T lymphocytes in the spleen appear within the white pulp by 1.4 hours. B lymphocytes take nearly 4.3 hours to achieve the same level of accumulation. In addition, T lymphocyte arrival is fucoidan sensitive, while B cells are not affected by this polysaccharide. Finally, we show that neither L-, E-, or P-selectin appears to play a significant role in the accumulation of lymphocytes in the white pulp.)

**ABBREVIATIONS:** HEV, high endothelial venule; PP, Peyer's patches; LN, lymph node; PNAd, peripheral node addressin; MAdCAM-1, mucosal addressin cell adhesion molecule-1; CFSE, 5-(and-6)-carboxyfluorescein diacetate, succinimidyl ester; TAMRA, 5-(and-6)-carboxytetramethylrhodamine, succinimidyl ester

### INTRODUCTION

The initial encounter of T cells with antigen is generally thought to occur in secondary lymphoid tissues. In a naïve host, this encounter requires a physical interaction between antigen-bearing, antigen-presenting cells and rare antigen-specific naïve T cells. The organized structure of the secondary lymphoid tissues is thought to facilitate these cellular interactions, as well as the interactions between T and B cells that underlie T cell help in the humoral response[1]. Identification of the molecular signals that control the establishment of normal cellularity of these tissues is important for understanding how initial T cell sensitization is regulated.

The mechanisms governing the recruitment of naïve lymphocytes to the lymph nodes (LN) and Peyer's patches (PP) have been generally well defined. In LN, adhesive cellular interactions at the high endothelial venule (HEV) control the initial movement of naïve lymphocytes out of the circulatory compartment into the organized lymphoid tissue. Naïve lymphocytes initially tether and role on peripheral node addressin (PNAd) expressed on the LN HEV[2]. The ligand for PNAd expressed on naïve lymphocytes is L-selectin (CD62L)[3]. Rolling lymphocytes can then adhere firmly via activation of their surface-expressed $\alpha L\beta 2$ integrin (CD11a/CD18; LFA-1) that interacts with intercellular adhesion molecule-1 (ICAM-1) on the HEV surface[4]. This activation is dependent upon binding of chemokines in the HEV glycocalyx to chemokine receptors on the surface of the rolling lymphocytes. For T cells, this is engagement of CCR-7 by CCL21 (SLC); for B cells it is engagement of CXCR-5 by CXCL13 (BLC/BCA-1)[5,6,7,8]. Firmly adherent lymphocytes then migrate across the HEV and, in an integrin-dependent manner, enter the lymphoid compartment of the LN before moving to the appropriate locations in the tissue[9].

The rules governing cell entry into the PP are generally similar, although the initial rolling and tethering is dependent more on the interaction of L-selectin with the counterligand mucosal addressin cell adhesion molecule-1 (MAdCAM-1)[10]. Subsequent firm adhesion is also dependent on MAdCAM-1 and its interaction with $\alpha 4\beta 7$ integrin (LPAM-1) on the lymphocyte[11,12].

In contrast, the rules governing movement of cells from the circulation into the lymphoid tissue compartment of the spleen are not well understood. The spleen is the single largest secondary lymphoid organ. Unlike the LN and PP that appear to function primarily in the presentation of antigens derived from epithelial tissues, the spleen is thought to facilitate immune recognition of antigens circulating in the blood. Lacking afferent lymphatic vessels, the structure of the splenic lymphoid compartment differs fundamentally from the LN and PP. In the spleen, the lymphoid compartment (the "white pulp") is surrounded by a reticuloendothelial compartment (the "red pulp")[13]. The lymphoid compartment consists of numerous nodules. Surrounding each nodule is a marginal zone that separates the white pulp from the red pulp[14]. Blood is thought to enter each white pulp nodule through a central arteriole that ends at a sinus within the marginal zone (the marginal sinus).

The pathways by which cells transit from these terminal arterioles to the red pulp and the venous vessels remains incompletely defined. Particularly, the site at which cells leave the circulation and enter the extravascular parenchyma of the spleen has not been fully defined. No HEV equivalent has ever been identified in the spleen. It has generally been presumed that the marginal sinus functions in this role[15]. This is due in part to the fact that the sinus lining cells on the white pulp side of the marginal sinus express on their surfaces MAdCAM-1[16], the cell adhesion molecule that is critical for the passage of circulating lymphocytes from the HEV into the lymphoid tissue parenchyma in the PP and mesenteric LN. However, MAdCAM-1 does not appear to play an essential role in the movement of cells from the circulation into the parenchyma of the spleen. This is based on the observation that none of the mouse strains deficient in one or more cellular adhesion molecules (including $\beta 7$ integrin or L-selectin, the two known ligands for

MAdCAM-1) has shown abnormal cellular localization in the spleen[3,11,17,18,19,20,21,22,23,24,25].

To understand more clearly the mechanisms controlling the movement of lymphocytes from the circulation into the white pulp of the spleen, we have examined the movement of adoptively transferred lymphocytes into the red and white pulp compartments. We find that adoptively transferred T lymphocytes accumulate in the white pulp more rapidly than do adoptively transferred B lymphocytes. In addition, while i.v. injection of fucoidan inhibits the accumulation of adoptively transferred T cells in the spleen, none of the known selectin molecules appears to play an essential role. Similarly, adoptively transferred B lymphoytes also find their way into the spleen independent of any of the known adhesion molecules.

### METHODS

#### Animal

C57BL6/J, B6/129, Rag-1 deficient, L-selectin deficient, and both E- and P-selectin deficient mice were obtained from Jackson Laboratories (Bar Harbor, ME) and were housed for at least 1 week prior to use in the animal barrier facility at Washington University School of Medicine. Mice used in these experiments were between 4 and 12 weeks old. Mice were housed in specific pathogen-free conditions with food and water provided *ad libitum*, and all experiments were approved by the Washington University institutional committee for the humane use of laboratory animals.

#### Polysaccharides and Antibodies

Rat IgM anti-MOMA-1 was obtained from Serotec, Inc. (Oxford, U.K.). Phycoerythrin (PE)-labeled anti-Rat IgM, biotin-labeled anti-Rat IgM, anti-CD16/32, unlabeled and biotin-labeled anti-Thy 1.2, biotin-labeled anti-$IgD^a$, biotin-labeled anti-Thy1.1, and unlabeled and biotin-labeled anti-B220 mAbs were all obtained from BD Pharmingen (San Diego, CA). Streptavidin-FITC, mannose, and fucoidan were obtained from Sigma Chemical Co. (St. Louis, MO). In some experiments mannose or fucoidan was injected with the lymphocytes i.v. Mannose was used at 20 mg/kg and fucoidan was given at 10 mg/kg.

#### Lymphocyte Isolation and Purification

T and B lymphocytes were isolated by negative selection using a MACS column as per the manufacturer's instructions (Miltenyi Biotec, Inc., Auburn, CA). For T cell isolation, anti-B220 and anti-CD16/32 mAbs were used with anti-Rat immunoglobulin conjugated to magnetic beads (Miltenyi Biotec). B lymphocytes were isolated using anti-CD43 magnetic beads (Miltenyi Biotec). Final populations were ≥90 % pure T cells or B cells as determined by flow cytometry.

### Adoptive Transfer of $^{51}$Cr-Labeled Cells

For $^{51}$Cr labeling, purified cells were incubated with 100 µCi $Na_2{}^{51}CrO_4$ (Amersham Pharmacia Biotech, Piscataway, NJ) for 60 min in media (complete RPMI with 10% fetal calf serum; Life Technologies, Rockville, MD). The cells were then washed several times in media and then resuspended in Dulbecco's PBS (Life Technologies). The cells were then aliquotted and injected i.v. via the retro-orbital plexus into recipient mice. An aliquot of each cell type was kept and used to determine the total amount of radioactivity injected. After 90 min for T cells and 4 h for B cells, the recipient mice were sacrificed and their internal organs and blood were removed. Radioactivity of the various organs (spleen, lungs, liver, heart, gastrointestinal tract, and kidneys) and the carcass was determined using a gamma counter. The percent radioactivity found in the tissues was calculated by dividing the counts in the tissue by the total counts injected into the mouse.

### Adoptive Transfer of Fluorescently Labeled Cells

Adoptive transfer studies were performed by incubating $5 \cdot 10^7$ cells/ml of purified T or B lymphocytes with 5 µM CFSE or 6 mg/ml TAMRA (both from Molecular Probes, Eugene, OR) for 15 min at 37$_o$C in the dark. Afterwards, the cells were washed twice and resuspended in sterile PBS (Life Technologies). Recipient mice were sedated by methoxyflurane (Schering-Plough Animal Health, Union, NJ) inhalation and $10_7$ cells in 100–150 µl PBS were then injected i.v. via the retro-orbital plexus. Mice were then humanely sacrificed by $CO_2$ inhalation at various time points and their spleens removed and frozen in O.C.T. Compound (VWR Scientific Products, So. Plainfield, NJ). Spleen sections (7-µM thick) were cut and stained for fluorescent histology as previously described[26].

### Analysis of Fluorescently Labeled Tissue Sections

Sections were examined using an Olympus BX-60 fluorescence microscope (Melville, NY). Sections at 100· magnification were photographed using a 3 chip cooled CCD camera (Optronics, Inc., Goleta, CA). The images were then imported into IPLab (Scanalytics, NY). Using this software the number of CFSE or TAMRA labeled cells were counted in each 100· field. In addition, the number of cells within the white pulp nodules (as marked by MOMA-1 staining) was determined. The percentage of transferred cells localizing within the white pulp nodules was then determined by dividing the number of cells in white pulp nodules per 100· field by the total number of cells in that 100· field. The data reported are the result of counting three or more 100· fields from three or more separate spleen sections from two mice per time point and cell type. Immunohistology was performed as previously described[27].

### Statistical Methods

Statistical analysis was performed using Student's T-test with significance set at $p < 0.05$.

## RESULTS AND DISCUSSION

### A Higher Fraction of Adoptively Transferred B Lymphocytes Localizes in the Spleen Compared to T Lymphocytes

In preliminary studies, we purified B and T cells from the spleens of unimmunized mice and labeled them with $^{51}$Cr prior to intravenous infusion back into naïve recipients. We harvested the spleens 1.5–2 h later, and measured the fraction of $_{51}$Cr that had accumulated in this tissue. We found that when $^{51}$Cr-labeled B cells were infused, 51 ± 12% of the injected $_{51}$Cr was recovered in the spleen, whereas when labeled T cells were infused, 15 ± 5% of the injected $^{51}$Cr was recovered (n = 2, mean ± SEM; these values were greater than twice that seen in any other tissue for both cell types). These data indicated that adoptively transferred B lymphocytes were nearly three times more likely to localize within the spleen than T cells.

### Adoptively Transferred T Lymphocytes Localize in the Spleen White Pulp More Rapidly than B Lymphocytes.

The preliminary experiments tracking $^{51}$Cr-labeled cells were useful for defining the efficiency with which adoptively transferred, purified cells localize within the spleen, but they were not informative regarding the ability of these cells to localize specifically within the white pulp. Under certain circumstances, adoptively transferred lymphocytes can accumulate in the spleen in normal numbers without entering the white pulp. For example, lymphocytes treated with pertussis toxin fail to gain entry into the white pulp, but do enter the spleen in normal numbers, localizing in the red pulp[28]. Therefore, in order to investigate the mechanisms governing the localization of T and B cells to the white pulp, we monitored the local accumulation of adoptively transferred cells using immunofluorescence microscopy.

In order to track the adoptively transferred T and B lymphocytes, we labeled the purified cell populations with fluorescent dyes, either 5-(and-6)-carboxyfluorescein diacetate, succinimidyl ester (CSFE) or 5-(and-6)- arboxytetramethylrhodamine, succinimidyl ester (TAMRA) as indicated. At the indicated times after i.v. adoptive transfer into naïve syngeneic recipients, the spleens were removed and frozen. Frozen sections were fixed and stained with the MOMA-1 monoclonal antibody, which identifies the metallophilic macrophages that are localized to the white pulp side of the marginal zone[29]. These stained sections were then analyzed by fluorescence microscopy to identify the numbers and locations of the adoptively transferred cells relative to the ring of MOMA-1$_{+}$ cells that marks each white pulp nodule. Digital images captured using a cooled-CCD camera were analyzed by the IP Lab software package to determine the numbers of adoptively transferred cells that were inside the MOMA-1–defined regions (white pulp) and outside (red pulp). For this analysis, a line was drawn manually through the center of the MOMA-1$^{+}$ area, and the region internal to that line was designated white pulp.

As seen in Fig. 1, adoptively transferred T cells were detected in the spleen as early as 30 minafter transfer. By 90 min after transfer, most of the T lymphocytes were localized within the white pulp nodules. There was little subsequent increase in the number of transferred cells accumulating within the white pulp compartment up to 18 h after transfer (see Fig. 3).

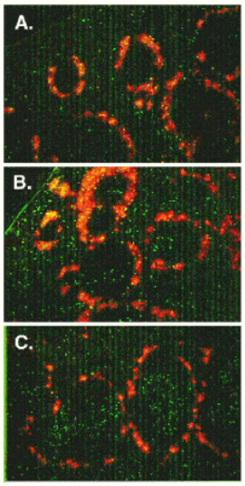

**FIGURE 1.** Time course of T lymphocyte accumulation in the white pulp of the spleen. $10^7$ purified T cells labeled with CFSE were injected into the retro-orbital plexus of C57BL/6 mice. Spleens were harvested 30 (panel A), 60 (panel B), and 90 (panel C) min after injection. The white pulp is delineated by the red staining of metallophilic macrophages using MOMA-1 (Rat IgM) bound to PE labeled anti-Rat IgM; the CFSE labeled cells appear green. Similar results were obtained in two additional experiments.

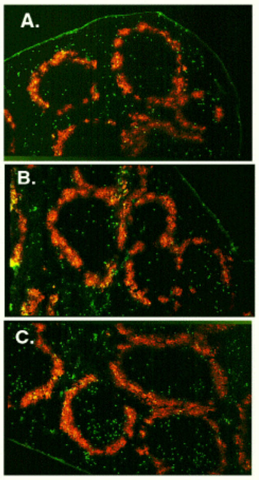

**FIGURE 2.** Time course of B lymphocyte accumulation in the white pulp of the spleen. $10^7$ purified B cells were treated as in Fig. 1. Spleens of recipient mice were harvested at 2 (panel A), 3 (panel B), and 4 (panel C) h after transfer. Spleens were frozen, sectioned, and stained as in Fig. 1. These images are representative of three similar experiments.

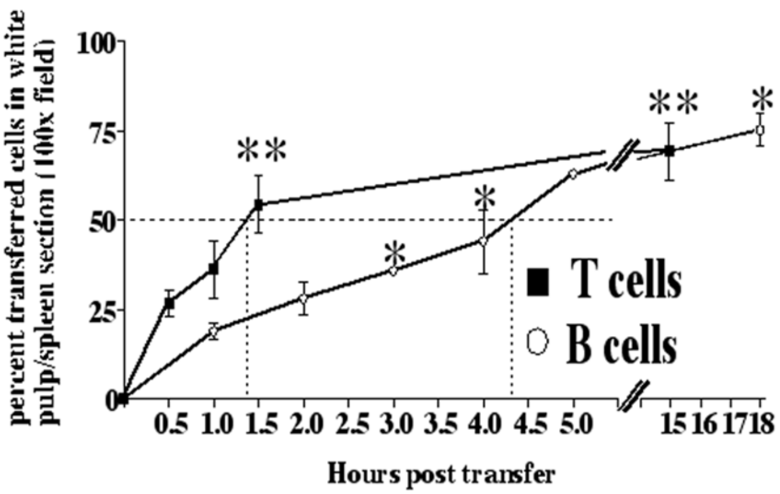

**FIGURE 3.** Comparison of T and B lymphocyte accumulation in the white pulp. The data represent the percent of transferred T cells or B cells per high powered field (100·) that are located within the MOMA-1–delineated white pulp. A minimum of three fields per spleen section, and a minimum of three spleen sections from two identically treated mice were analyzed for each time point for each cell type (mean ± SEM). The time at which 50% of the transferred cells in the spleen were located within the white pulp is indicated by the dotted line. Note that for T lymphocytes this is 1.4 h, whereas for B cells this is 4.3 h. * $p < 0.05$ vs. 1 h B cell timepoint; ** $p < 0.05$ vs. 30 min T cell timepoint.

Adoptively transferred B cells were also detected within the spleen shortly after i.v. infusion, although they were modestly delayed compared to T cells. Indeed, while T cells reached their maximum accumulation in the spleen within 1 h of transfer, it was not until 2 h after i.v. infusion that B cells reached their maximum accumulation within this organ (data not shown). Most striking, however, was the length of time required for B cells to gain entry into the white pulp nodules. As shown in Fig. 2, significant accumulation of B cells in the lymphoid compartment was not detected until 2 h after adoptive transfer, and it was not until just over 4 h that greater than half of the transferred B cells in the spleen were localized within the lymphoid compartment. At further time points up to 18 h after transfer, there was little increase in the fraction of splenic B cells partitioning into the white pulp.

A comparison of the accumulation of T and B cells in the white pulp is shown in Fig. 3. Note that the point at which 50% of the transferred cells in the spleen appear within the white pulp is approximately 1.4 h for T lymphocytes and approximately 4.3 h for B lymphocytes. By 18 h after adoptive transfer, for both T and B cells, approximately 70% of the adoptively transferred cells were localized in the white pulp.

Because of concerns that the fluorescent dyes used in these experiments might disturb normal cellular migratory behavior, we repeated selected experiments using transfer of unlabeled C57BL/6 T and B cells into RAG-$1^{-/-}$ mice. In other experiments, we used transfer of unlabeled lymphocytes from Thy1.1/$IgD^a$ donors into Thy1.2/$IgD^b$ recipients. Localization of the transferred B and T cells was determined by immunohistochemical staining with anti-B220, anti-Thy1.2, anti-$IgD^a$, and anti-Thy1.1. The experiments confirmed the different time courses under which T cells and B cells entered the spleen white pulp (data not shown).

The fact that the time courses for B and T cell entry into the white pulp were different suggested that the mechanisms regulating the movement of B and T cells from the blood circulation to the lymphoid compartment were different. It has generally been assumed that the marginal sinus represents the lymph node HEV equivalent in the spleen. Of interest, although we detected cells in both the red and white pulp compartments, few cells were detected in close proximity to the marginal sinus. Thus, we anticipate that sites other than the marginal sinus may be the primary locations for lymphocyte entry into the lymphoid compartment of the spleen.

### Migration of T Lymphocytes to the Spleen White Pulp is Inhibited by Fucoidan

Fucoidan is a sulfated poly-L-fucose compound that is produced by the fungus *Fucus vesiculosus*. It has been shown to block selectin-mediated lymphocyte migration in the rat mesentery[30] and to interfere with accumulation of adoptively transferred lymphocytes into the white pulp of the spleen[31,32]. However, these experiments did not localize the adoptively transferred cells relative to the white pulp architecture[31,32]. We extended these preliminary studies to test whether fucoidan altered the localization of purified T or B cells relative to the defined elements of the lymphoid compartment of the spleen. As shown in Fig. 4, coinjection of fucoidan together with purified T cells led to a greater than 65% reduction in the accumulation of the adoptively transferred cells in the white pulp compartment. Coinjection of mannose as a control for the osmotic effects of fucoidan did not induce a statistically significant alteration in T cell accumulation in the white pulp. This is consistent with the prior findings of Weston and Parish that mannose does not alter the distribution of adoptively transferred mixed T and B cells in the spleen[31].

While our experiments using adoptive transfer of purified T lymphocytes confirmed the initial findings of Brenan and Parish[32], we found no effect of fucoidan on B cell accumulation in the white pulp (data not shown). Brenan and Parish did not directly study the effect of fucoidan on B cells. Their studies did imply, however, that fucoidan had a stronger effect on T cells than B cells based on analysis of white pulp histology[32]. Additionally, we used a 20-fold lower dose of fucoidan

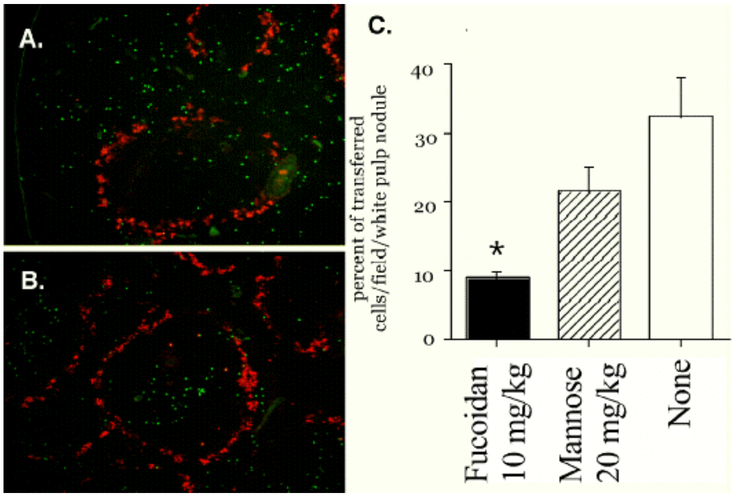

**FIGURE 4.** Fucoidan inhibits T lymphocyte accumulation in the white pulp of the spleen. $10^7$ purified C57BL6/J CFSE-labeled T lymphocytes were injected into syngeneic recipients together with either 10 mg/kg fucoidan (panel A) or 20 mg/kg mannose (panel B). Spleens were harvested 90 min after transfer and stained as in Fig. 1. Panel C shows that the only significant difference between untreated, fucoidan-treated, and mannose-treated lymphocytes was that the fucoidantreated T lymphocytes accumulated less in the white pulp than untreated cells. Mannose had no significant effect on cell accumulation. The number of transferred cells within the white pulp was calculated as in Fig. 3. Data are presented as means ± SEM; n > 3 mice per timepoint. *$p < 0.05$ vs. lymphocytes transferred without fucoidan or mannose.

than that used in the previous studies (in our studies, 10 mg/kg = approximately 0.2 mg/mouse vs. 4–5 mg/mouse in the previously published studies). Our finding that infusion of fucoidan did not alter the accumulation of B cells in the white pulp may reflect the possibility that the B cell spleen targeting mechanism is less sensitive to fucoidan than that of T cells. Alternatively, the apparent lack of activity of fucoidan on B cell accumulation in the spleen may reflect the possibility that the actions of fucoidan have dissipated by the time that B cells begin to accumulate in significant numbers. There is support for this possibility in the literature, with the *in vivo* effects of fucoidan waning by 3 h after i.v. infusion in a rabbit model of experimental meningitis[33]. This latter possibility is less likely, because in a single experiment we administered fucoidan two times (first, together with the B cells, and then second, 1.5 h later). Again, fucoidan did not appear to have an effect on B cell accumulation (data not shown). Lastly, it remains fully possible that the mechanisms governing B lymphocyte entry into the lymphoid compartment of the spleen are distinct from those governing entry of T lymphocytes and are not fucoidan sensitive.

### Accumulation of B and T Lymphocytes in the Spleen White Pulp is Not Dependent on L-, E-, or P-Selectin

Since fucoidan is a sulfated poly-L-fucose and selectin molecules bind sulfated poly-L-fucose through their recognition domains, we examined the potential role that the known selectin molecules might play in accumulation of adoptively transferred lymphocytes within the white pulp. In LN and PP, it has been shown that expression of L-selectin on the lymphocyte is important for rolling and tethering on the surface of the HEV[9]. In addition to L-selectin, the selectin family of molecules includes P- and E-selectin. These latter two molecules are expressed on the surfaces of endothelial cells, and in the case of P-selectin, on the surfaces of platelets[34]. In order to examine the potential role of all known selectin molecules on the accumulation of adoptively transferred lymphocytes in the spleen, we analyzed the behavior of lymphocytes harvested from mice deficient in one, two, or three of the known selectin molecules[35]. In the initial characterization of these gene-targeted mice, there has been no recognized reduction in the cellularity of the spleen white pulp. In contrast, L-selectin deficient mice manifest an increase in total spleen size[3]. This has been thought to represent a compensatory increase in lymphocyte accumulation in the spleen as a consequence of inability of lymphocytes to localize efficiently to the LN and PP in L-selectin deficient animals.

It is important to appreciate that the retention of apparently normal splenic white pulp cellularity in mice deficient in the known selectin molecules does not exclude participation of selectins in localization of lymphocytes within this lymphoid organ. Selectin molecules might normally play a major role in the initial localization of lymphocytes in the spleen parenchyma, but in the their absence other, perhaps less efficient, molecular mechanisms might be recruited to permit cellular entry into this tissue. If this were the case, we hypothesized that the time course of accumulation of selectin-deficient lymphocytes to the white pulp might be altered compared to that of wild type lymphocytes.

To determine if selectin molecules do indeed play a role in lymphocyte accumulation to the white pulp, we analyzed the behavior of adoptively transferred T and B cells from L-selectin deficient donors in recipients that were deficient for both P- and E-selectin. We analyzed the

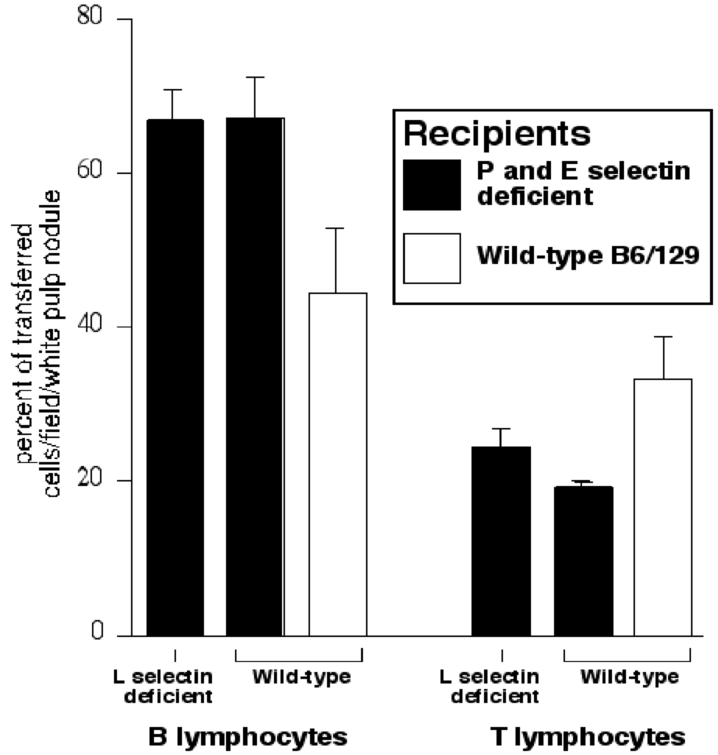

**FIGURE 5.** Accumulation of T and B lymphocytes in the lymphoid compartment of the spleen does not depend upon L-, E-, or P-selectin. $10_7$ fluorescently labeled C57Bl6 · 129 (B6/129, wild-type) or L-selectin deficient T or B lymphocytes were injected into B6/129 or P- and E-selectin deficient recipients. Spleens were harvested after 90 min for T cells and after 4 h for B cells. The number of transferred cells located within white pulp nodules was determined as in Fig. 3. Regardless of the donor or recipient, there was not a significant difference in the total number of cells (counting those both within and outside the white pulp nodules) found in each 100· field for both T and B lymphocytes (data not shown). Note also that there was no significant difference in the accumulation of lymphocytes in the white pulp between the selectindeficient and the wild-type mice. Data are presented as means ± SEM; n > 3 mice per timepoint.

numbers of T and B cells in the white pulp at 90 min and 4 h, respectively, in order to maximize the likelihood of observing altered cellular accumulation in the case that the rate of cell entry into the white pulp was altered. As shown in Fig. 5, there were no significant decreases in the numbers of T or B lymphocytes accumulating in white pulp nodules when L-selectin deficient cells were compared to wild-type T and B lymphocytes after transfer into wild-type recipients. Nor was there a decrease in the number of cells appearing within the white pulp nodules when L-selectin deficient cells were transferred into E- and P-selectin deficient hosts. These data suggest that the selectin molecules do not contribute in measurable fashion to the initial localization of lymphocytes to the white pulp of the spleen. Interestingly, there was a small but reproducible increase in the accumulation of both wild type and L-selectin deficient B lymphocytes in the white pulp after transfer into E- and P-selectin doubly deficient hosts compared to transfer into wild-type hosts. While this increase did not reach statistical significance, it did suggest that E- and P-selectins may be important in the homing of B lymphocytes to compartments other than the spleen, with their absence leading to secondary alterations in the accumulation of B cells in the white pulp. Further investigations will be necessary to clarify this observation.

We have shown that T and B lymphocytes accumulate in the splenic white pulp with differing time courses, and that only T lymphocyte migration can be inhibited by treatment with fucoidan (10 mg/kg). This suggests that T and B cells differ in the molecular mechanisms that control their accumulation in this lymphoid tissue. Further we show that the known selectin molecules, L-, E-, and P-selectin, do not play a significant role in this tissue localization.

### ACKNOWLEDGEMENTS

We would like to thank Carlene Zindl, Laura Mandik-Nayak, and Craig Byersdorfer for their helpful discussions and encouragement.

This research was supported by a Barnes-Jewish Foundation grant (to MHG) and a National Institutes of Health grant (AI-34580 to DDC). During the period in whixh this work was performed, D.D.C. was an investigator of the Howard Hughes Medical Institute.
